# Does complexity matter? Meta-analysis of learner performance in artificial grammar tasks

**DOI:** 10.3389/fpsyg.2014.01084

**Published:** 2014-09-25

**Authors:** Rachel Schiff, Pesia Katan

**Affiliations:** ^1^Learning Disabilities Studies and Haddad Center for Dyslexia and Learning Disabilities, School of Education, Bar-Ilan UniversityRamat-Gan, Israel; ^2^Learning Disabilities Studies, School of Education, Bar-Ilan UniversityRamat-Gan, Israel

**Keywords:** artificial grammar learning, topological entropy, complexity, grammar system

## Abstract

Complexity has been shown to affect performance on artificial grammar learning (AGL) tasks (categorization of test items as grammatical/ungrammatical according to the implicitly trained grammar rules). However, previously published AGL experiments did not utilize consistent measures to investigate the comprehensive effect of grammar complexity on task performance. The present study focused on computerizing Bollt and Jones's (2000) technique of calculating topological entropy (TE), a quantitative measure of AGL charts' complexity, with the aim of examining associations between grammar systems' TE and learners' AGL task performance. We surveyed the literature and identified 56 previous AGL experiments based on 10 different grammars that met the sampling criteria. Using the automated matrix-lift-action method, we assigned a *TE* value for each of these 10 previously used AGL systems and examined its correlation with learners' task performance. The meta-regression analysis showed a significant correlation, demonstrating that the complexity effect transcended the different settings and conditions in which the categorization task was performed. The results reinforced the importance of using this new automated tool to uniformly measure grammar systems' complexity when experimenting with and evaluating the findings of AGL studies.

## Introduction

Artificial grammar learning (AGL) refers to an experimental approach that explores pattern recognition in a set of structured sequences, typically comprising strings of alphabetical letters. Such experiments include a training phase and a testing phase (Reber, 1967, 1969). Studies on AGL have demonstrated that participants are able to acquire the abstract representation or rule underlying an artificial grammar system.

Researchers have utilized AGL tasks to explore the distinction between explicit and implicit learning, to identify the representations acquired through learning, and as a model for the process of language acquisition. AGL studies can be carried out either explicitly, where the participant is informed during the training phase that the stimuli were constructed according to a set of rules, or implicitly, where the participant is unaware of the fact that certain rules underlie the stimuli (Reber, 1967; Lorsbach and Worman, 1989; Gebauer and Mackintosh, 2007). Various theories have been debated to explain what characterizes the learning process and what is acquired during AGL training sessions, including the probabilistic learning approach (Reber, 1967), the exemplar-based learning approach (Brooks and Vokey, 1991), and a third approach suggesting that learners' acquisition of abstract rules during the training phase enables them to judge the sequences at the testing phase (Redington and Chater, 1996; Pothos, 2010). The AGL paradigm has also been proposed as a model for language or syntax acquisition, but questions remain as to how broadly it applies to the tasks faced by language learners (Marcus et al., 1995; Pena et al., 2002; Endress and Bonatti, 2007; Aslin and Newport, 2008).

AGL experiments typically involve letter strings that are generated based on a grammar system and then are shown to the learner during a training session. In studies of implicit learning, learners are asked to memorize stimulus strings during this training phase (Dienes et al., 1991; Pavlidou et al., 2009), whereas in explicit AGL tasks, learners are informed during training that the stimuli were constructed according to a complex set of rules, and they are asked to look for those rules (Kirkhart, 2001; Gebauer and Mackintosh, 2007). In the testing phase, two types of strings, grammatical and ungrammatical, are shown to learners. Learners are asked to decide whether or not the strings are grammatical, namely, whether they were constructed using the same set of rules used to construct the strings shown in the training session (Reber, 1967). Participants consistently score above chance on such AGL tasks, even when they are unable to specifically extract the underlying rule and regardless of whether learners were initially informed explicitly about strings' rule-based construction (Dulany et al., 1984; Dienes et al., 1991). This consistent finding in the literature indicates that individuals are able to acquire some awareness of the rule even without explicit exposure to the rule's existence (Servan-Schreiber and Anderson, 1990; Gebauer and Mackintosh, 2007).

**Figure 1:**
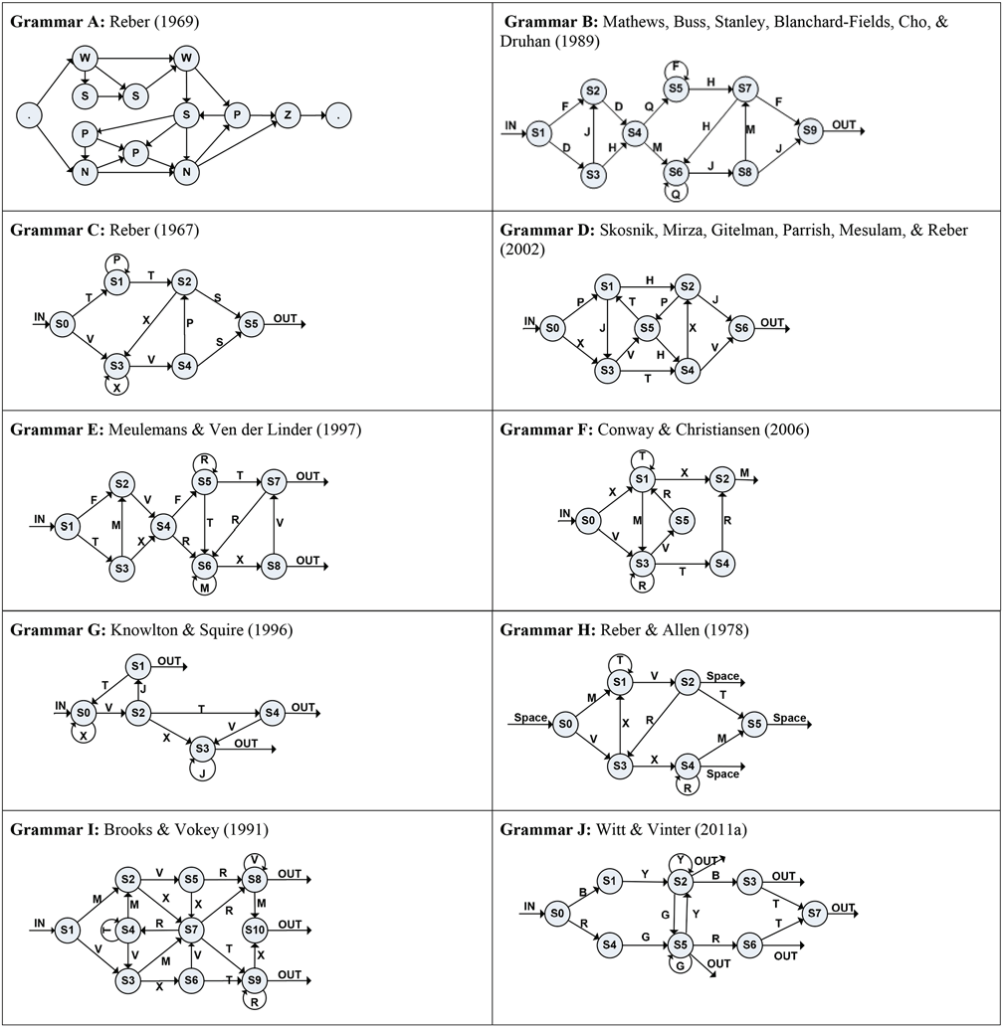
**Charts of the 10 artificial grammars appearing in Table 1**.

The standard AGL experiment is based on a grammar system, with sequences of symbols presented as letter strings such as TTS or VXVPS (Reber, 1967). For example, when using Reber's (1967) grammar chart (see Grammar C in Figure 1), all sequences must begin at the IN arrow and end at the OUT arrow. The path from the IN arrow can be either from State 0 (S0) to State 1 (S1) or from S0 to State 3 (S3). Therefore, the first symbol can only be a T or a V. Moving on, if a sequence begins with a V, then the only possible transitions are from S3 to itself (see curved arrow in the figure), thereby adding an X, or else from S3 to S4, thereby adding a V. If a second V is added after the X by moving to S4, then we can next move in one of two directions: from S4 to S2, adding a P, or from S4 to S5, adding an S and ending the sequence. In contrast, from S4 the move back to S3 is illegal (ungrammatical); therefore, adding a consecutive V is not an option. Thus, using this chart, a sequence such as VXVS is grammatical, but creating a sequence such as VXVV is ungrammatical (Pothos and Kirk, 2004).

Previous AGL studies have come under heavy criticism for the effect of grammar system complexity on learners' performance (Reber, 1967; Perruchet et al., 1998; Van den Bos and Poletiek, 2008), claiming that when the grammar system includes more rules (arrows), uncertainty increases, and the grammar is harder to acquire, and vice versa (Ziegler and Goswami, 2005). However, complexity has not always been systematically defined by AGL researchers, who have employed different ways to measure the complexity of AGL stimuli's structure.

Johnstone and Shanks (2001) suggested one method for measuring complexity of a testing item, by calculating its “transition rule strength” (TRS), which refers to the number of rules constituting the grammar system. These rules are defined as (a) the number of letters that can be added to a string at each node of the grammar chart and (b) the number of letters that can terminate a string. For example, using Brooks and Vokey's (1991) chart (see Grammar I in Figure 1), Johnstone and Shanks calculated a total of 17 transitions (arrows), including 3 nodes where the same letter could be used more than once (S2, S3, S4) and 3 nodes where the string could end (S8, S9, S10). Johnstone and Shanks (1999) asserted that more repetitions of a specific transition from one node to another (e.g., the transition from S7 to S8, adding the letter R, in Grammar I) during the entire training phase would lower that particular rule's complexity. To calculate this complexity measure for a particular item on a testing task (where participants were asked to indicate if a given string was grammatical), these researchers summed the number of times each transition in the testing string had appeared across all of the trained strings, and then they divided that sum by the number of letters forming that specific testing string. For example, using Brooks and Vokey's grammar (see Grammar I in Figure 1), the testing string VXVRM contained the following six transitions: V was selected at the transition from S1 to S3, X at the transition from S3 to S6, V at the transition from S6 to S7, R at the transition from S7 to S8, M at the transition from S8 to S10, and the ending transition was at S10. In Johnstone and Shanks' (1999) training phase, these six transitions had appeared 64, 33, 17, 45, 19, and 39 times respectively, across 125 training strings. The calculation of complexity for VXVRM was therefore (64 + 33 + 17 + 45 + 19 + 39) divided by 5 letters, totaling 43.4. They concluded that increasing the number of transitions increased the string's complexity (Johnstone and Shanks, 1999, 2001).

A second way to measure the complexity of a test item was proposed by Perruchet et al. (1998), who noted that learning, may be influenced by the number of “segments” to be acquired. In other words, when presented with information that contains multiple elements, segmentation into smaller units leaves larger but fewer units or “chunks” to remember (e.g., 2-letter bigram chunks or 3-letter trigram chunks). Moreover, a bigram that repeats itself in the stimuli creates a simpler structure than one composed of many unique bigrams (Van den Bos and Poletiek, 2008). For each test item (string), a “global chunk strength” value can be calculated, comprising the average frequency at which all of its bigrams and trigrams appeared during the training phase (Knowlton and Squire, 1994, 1996). For example, in computing the chunk strength of testing string MSXVVR, one must calculate how frequently each of the following chunks appeared in training—MS, SX, XV, VV, VR, MSX, SXV, XVV, and VVR—and calculate their average (Pothos, 2010).

A third means of measuring the complexity of AGL stimuli, the “exemplar” view, derives from information theory. Jamieson and Mewhort (2005) proposed that intact stimuli are stored in memory, and that classification or recognition is determined by the degree of similarity between a stimulus and the stored exemplars. They explored the effect of redundancy on implicit learning, Jamieson and Mewhort quantified the structure in individual stimuli (local redundancy) as well as the structure in the grammatical rules from which the exemplars were derived (grammatical redundancy). The two kinds of redundancy were found to be correlated, where local redundancy increased with grammatical redundancy. However, when separated experimentally, performance was predicted by local redundancy but not by grammatical redundancy.

We assert that these varying measures of complexity adopted by prior researchers may preclude reliable comparison and interpretation of the mixed findings yielded by previous AGL research. In the current article, we propose Bollt and Jones's (2000) topological entropy (TE) measure of complexity as the tool of choice to enable quantitative standardization of the different grammatical charts on which all AGL system stimuli are based. Each AGL chart consists of nodes (states) and arrows showing the transitions' directional flow. Bollt and Jones (2000) developed the concept of TE by showing that each chart can be represented by a transition matrix (also known as Markov matrix), which is a mathematical way of representing all possible transitions in a given chart. Raising the number of transitions (arrows) increases TE, thus increasing uncertainty, and vice versa. The TE measure is also sensitive to the size of the matrix that represents a given chart and to the presence of short- and long-distance dependencies, and it is also correlated with the number of elements required to determine the current state in the chart. The formal calculation of TE as presented in definition eight in Bollt and Jones (2000) is described below:

$$h\left(\sum_{M}\right)=\underset{n\to \infty }{\lim}\;\left[\frac{\ln\left(w_{n}\left(\sum_{M}\right)\right)}{n}\right]$$

In this formula, *h*(∑_*M*_), which is the TE of ∑_*M*_, is defined to be the limit of the logarithm of the number of words of length *n*, divided by *n*, as *n* goes to infinity. More specifically, *M* is a transition matrix of a Markov representation (defined by the AGL chart), and ∑_*M*_ is the set of all possible sequences defined by *M*. Also, *w*_*n*_(∑_*M*_) is the number of sub-sequences of length *n* that are contained in ∑_*M*_, and *n* is the length of a single sequence. Note that although the length of a single sequence is one of the parameters in the formula, the TE formula has no dependency on length. The formula is not meant to calculate the results for a specific sequence, but rather to give a global index that measures the complexity of the chart. Bollt and Jones (2000) also presented a useful technique for calculating the TE of a given transition matrix of a Markov representation. Theorem 6 in Bollt and Jones (2000) states that if ρ(*M*) is the largest non-negative eigenvalue of the matrix *M*, then *h*(∑_*M*_) = ln ρ(*M*).

**Figure 2:**
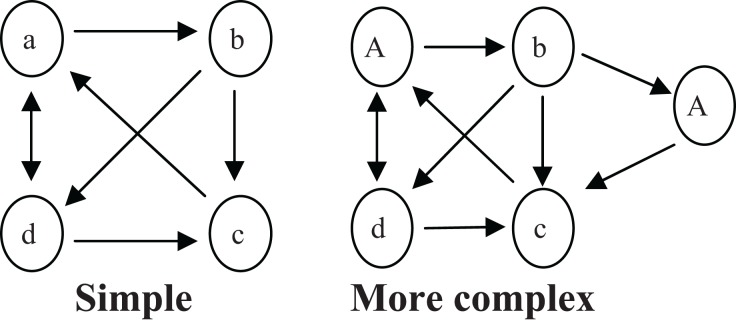
**A simple grammar chart and a more complex grammar chart**.

Computing TE directly from the formula can be very difficult. Bollt and Jones (2000) explained that in complex cases when an element appears more than once, a simple matrix cannot represent the chart. Figure 2 presents examples of a simple and a complex chart. When constructing the complex chart, knowing which letter in the sequence precedes A is the key to identifying at which A we are positioned. Bollt and Jones (2000) defined this as a system with memory (of the previous element or elements). However, a Markov matrix representation is restricted because the system it represents must be memoryless; that is, the transition to the next element must depend only on the current element without needing any reference to prior or ensuing transitions. For that reason, Bollt and Jones (2000) developed the concept of “lift action,” defined as a change in the dimension of the matrix required to build a memoryless system. Bollt and Jones presented a calculation of the lift action for the complex chart in Figure 2. They used the letter **m** to define the basic number of elements (excluding repeated elements), which in the case of Figure 2's complex chart would be **m** = 4 (a, b, c, and d). They used the letter **k** to define the minimum number of elements required in order to know one's present position in the chart. In the complex chart (Figure 2), **k** would equal 2, indicating that at least two transitions are required to know at which A in the chart we are positioned. In order to make a lift, Bollt and Jones defined *m*^*k*^ new elements based on the original elements. The new elements will be all the possible sequences in length **k** of the **m** original elements. In the present example, the minimum number of transitions required to identify at which A we are located is two (i.e., the number of basic elements that every new element will contain after the lift action is **k** = 2). The number of new elements will be *m*^*k*^, namely 16: aa = 1, ab = 2, ac = 3, ad = 4, ba = 5… The matrix will now be at size *m*^*k*^ × *m*^*k*^ (16 = 4^2^ in the current example, i.e., the matrix will have 16 × 16 options), which will include all possible transitions according to the chart's arrows, where each transition is represented by two elements.

In general, when elements in the chart are repeated more often, a larger sequence of elements needs to be remembered in order to determine one's current state in the chart, thereby increasing memory load. Hence, these types of charts cannot be represented in a simple way. Bollt and Jones (2000) demonstrated that a chart's TE is easily extracted as the natural logarithm of the largest positive real eigenvalue of the matrix; however, in a large matrix the eigenvalue must be calculated by a computer. Thus, Bollt and Jones's method for calculating TE is much more practical than computing TE directly from the formula, as explained in Appendix A in Supplementary Material.

In the current article, we expanded on Bollt and Jones's (2000) matrix-lift-action method by suggesting a novel technique for automating the full process of determining a specific grammar's complexity level. As explained in detail in Appendix B in Supplementary Material, we extended the code used in Bailey and Pothos's (2008) StimSelect software to uniformly calculate grammatical complexity for various AGL charts used in many prior research studies, thus enabling meta-analysis of learner performance in previous investigations of artificial grammar tasks based on different grammars. Once the level of complexity of each prior grammar chart was assessed, the following question was addressed: Did ____er *TE* values correlate with greater learner success (identifying grammatical/ungrammatical strings above chance level—over 50%) in AGL studies? We also examined the effect of age in AGL task performance, to determine if age differences between children and adults would transcend the effects of grammatical complexity.

## Materials and methods

### Meta-analysis literature survey

To systematically locate the maximum possible number of charts representing AGL systems that appeared in previously published experimental research, we conducted a comprehensive review of the AGL empirical literature. We used different databases to find the articles: Google, Google Scholar, ProQuest, PubMed, and APA PsycNET. The keywords selected for the literature review were: artificial grammar learning, topological entropy, and explicit and implicit learning. We looked only for studies written in English or translated into English. We searched for articles written after 1967 (following Reber's first article from that year). We included mostly journal articles but also a few book chapters. Inclusion criteria for the literature we identified were as follows:

1. Articles that did not include an experiment, such as review articles, were excluded. Review articles usually present other researchers' studies or do not present experiments at all (e.g., Reber, 1989; Cleeremans et al., 1998).
2. We excluded studies that did not include and describe a visual chart of the finite state grammar used (e.g., Saffran et al., 1996, 1997). Thus, auditory-based AGL studies (e.g., Andrade and Baddeley, 2011; Rohrmeier et al., 2011) were not included because many did not include a chart, and their findings were inconclusive as to associations between the visual and auditory paradigms.
3. Studies that developed stimuli from a combination of two or more different finite state grammars were also excluded, as we were unable to determine to which of the charts the results could be attributed (e.g., Knowlton and Squire, 1994; Reber and Squire, 1999; Van den Bos and Poletiek, 2008).
4. In articles including more than one experiment, one of which was the classic version of the AGL task and the other a manipulation of the stimuli, only the classic version was included. For example, we excluded Jamieson and Mewhort's (2010) manipulated version, which included blanks between the letters which the participants were requested to fill. In Evans et al. (2009), we excluded the second version of the experiment that required participants to choose an ice cream flavor according to the sequences they learned in the training phase. We only used the version that implemented the classic AGL task to avoid confounding variables related to changes in procedures.
5. In articles examining special populations, for example persons with dyslexia (Rüsseler et al., 2006), Williams' Syndrome (Don et al., 2003), or other atypically developing individuals, we included only the results of the control group because the present study explored typical populations only.
6. In multisession AGL articles (e.g., Reber, 1967; de Vries et al., 2010), only the results of the first session were included to avoid confounding variables related to multisession procedures.
7. With respect to articles that utilized both explicit and implicit instructions (Gebauer and Mackintosh, 2007; Scott and Dienes, 2008), only the results of the implicit stimuli were used to avoid inconsistencies between the results of these two methods. Furthermore, as relatively fewer studies involved explicit learning, comparison was impossible.
8. For a few AGL charts (e.g., Gebauer and Mackintosh, 2007), calculating the TE according to Bollt and Jones's (2000) matrix-lift-action technique was beyond our ability, because we could not find a memoryless system for these charts, even after doing a lift with *k* = 10. We do not know the implications of excluding these extremely complex charts from our meta-analysis. Inasmuch as we could not evaluate them according to the currently proposed theory, we could not draw conclusions about how such complexity levels may influence participants' task performance.
9. Articles with different kinds of visual stimuli (e.g., letters, shapes, colors) were included in the analysis because Pothos et al. (2006) showed that the type of visual stimuli presented to participants demonstrated no significant effects on performance.

Based on these inclusion criteria, we located 56 experiments deriving from 38 publications as presented in Table 1, referring to 10 different grammatical charts (see Figure 1).

**Table 1:** **Experimental AGL studies by grammar's chart, *TE* value, and No. of stimuli and participants' number, age, and task performance accuracy**.

	**Publication**		**AGL grammar**			**Participants**		
**No**.	**Researchers**	**Year**	**Chart**	***TE***	**No. of stimuli**	**Sample/*M* age in years**	***N***	**Accuracy in %**
1.	Scott and Dienes	2008	A: Reber1969	0.560	45	22	40	69.00
2.	Kirkhart	2001	A: Reber1969	0.560	75	Students	12	61.40
3.	Domangue et al.	2004	B: Mathews1989	0.578	88	Students	46	70.00
4.	Reber	1967	C: Reber1967	0.602	28	Students	5	73.50
5.	Reber	1967	C: Reber1967	0.602	28	Children	5	65.20
6.	Zizak and Reber	2004	C: Reber1967	0.602	60	Students	30	62.00
7.	Zizak and Reber	2004	C: Reber1967	0.602	60	Students	29	62.00
8.	Zizak and Reber	2004	C: Reber1967	0.602	60	Students	25	58.00
9.	Zizak and Reber	2004	C: Reber1967	0.602	60	Students	34	59.00
10.	Servan-Schreiber and Anderson	1990	C: Reber1967	0.602	20	Students	9	68.90
11.	Rosas et al.	2010	C: Reber1967	0.602	16	7.31	15	47.00
12.	Poznanski and Tzelgov	2010	C: Reber1967	0.602	160	Students	12	67.50
13.	Conway and Christiansen	2006	C: Reber1967	0.602	54	Students	10	62.00
14.	Skosnik et al.	2002	D: Skosnik2002	0.603	50	Students	23	65.10
15.	Skosnik et al.	2002	D: Skosnik2002	0.603	50	Students	23	57.40
16.	Peigneux et al.	1999	E: Meulemans1997	0.686	51	63	17	56.13
17.	Danion et al.	2001	E: Meulemans1997	0.686	51	33.7	14	74.80
18.	Conway and Christiansen	2006	F: Conway2006	0.716	54	Students	10	66.00
19.	Conway and Christiansen	2006	F: Conway2006	0.716	54	Students	10	58.00
20.	Pavlidou et al.	2009	G: Knowlton1996	0.740	69	6.48	16	59.37
21.	Knowlton and Squire	1996	G: Knowlton1996	0.740	46	63.8	18	63.50
22.	Chang and Knowlton	2004	G: Knowlton1996	0.740	46	Students	30	62.50
23.	Chang and Knowlton	2004	G: Knowlton1996	0.740	46	Students	35	64.70
24.	Don et al.	2003	G: Knowlton1996	0.740	48	23	27	66.00
25.	Pavlidou et al.	2010	G: Knowlton1996	0.740	8	9.3	16	55.00
26.	Pavlidou and Williams	2010	G: Knowlton1996	0.740	8	Children up to 12	16	60.00
27.	Pothos and Kirk	2004	G: Knowlton1996	0.740	69	Students	74	49.00
28.	Pothos et al.	2006	G: Knowlton1996	0.740	60	Students	10	67.20
29.	Pothos et al.	2006	G: Knowlton1996	0.740	60	Students	10	64.80
30.	Horan et al.	2008	G: Knowlton1996	0.740	46	18–25	43	58.00
31.	Pothos and Bailey	2000	G: Knowlton1996	0.740	69	Students	24	59.00
33.	Pothos et al.	2006	H: Reber1978	0.761	60	Students	20	55.40
33.	Pothos et al.	2006	H: Reber1978	0.761	60	Students	20	53.30
34.	Pothos	2005	H: Reber1978	0.761	40	Students	16	61.87
35.	Rüsseler et al.	2006	H: Reber1978	0.761	20	32.75	12	64.10
36.	Perruchet and Pacteau	1990	H: Reber1978	0.761	20	Students	30	63.30
37.	Reber and Perruchet	2003	H: Reber1978	0.761	20	Students	15	55.60
38.	Reber and Perruchet	2003	H: Reber1978	0.761	20	Students	15	56.20
39.	Reber and Perruchet	2003	H: Reber1978	0.761	20	Students	20	55.30
40.	Smith et al.	2001	H: Reber1978	0.761	23	68.36	14	57.90
41.	de Vries et al.	2009	H: Reber1978	0.761	100	22.6	20	66.40
42.	Jamieson and Mewhort	2010	H: Reber1978	0.761	20	Students	39	60.00
43.	Jamieson and Mewhort	2010	H: Reber1978	0.761	40	Students	47	63.00
44.	Knowlton and Squire	1994	I: Brooks1991	0.856	16	62.4	6	62.50
45.	Knowlton and Squire	1994	I: Brooks1991	0.856	16	64.9	11	60.90
46.	Meulemans and Van der Linden	1997	I: Brooks1991	0.856	16	Students	20	55.60
47.	Meulemans and Van der Linden	1997	I: Brooks1991	0.856	32	Students	20	54.10
48.	Higham	1997b	I: Brooks1991	0.856	32	Students	20	58.00
49.	Gebauer and Mackintosh	2007	I: Brooks1991	0.856	60	11–32	103	66.42
50.	Tunney	2005	I: Brooks1991	0.856	32	19.42	42	58.00
51.	Newell and Bright	2003	I: Brooks1991	0.856	80	Students	30	51.30
52.	Higham	1997a	I: Brooks1991	0.856	48	Students	24	64.00
53.	Higham	1997a	I: Brooks1991	0.856	48	Students	40	50.00
54.	Higham	1997a	I: Brooks1991	0.856	48	Students	40	52.00
55.	Witt and Vinter	2011a	J: Witt2011	0.916	48	5–7	40	50.00
56.	Witt and Vinter	2011b	J: Witt2011	0.916	48	5–7	10	50.00

*AGL, artificial grammar learning. The different grammar charts (A through J) can be seen on Figure 1. TE, topological entropy. Number of stimuli = amount of test items (strings). Students = university students. The 10 charts are presented in ascending order of complexity*.

### Procedure

As described in detail in Appendix B in Supplementary Material, we selected and developed software to fully automate TE calculation from a given AGL chart. We selected Bailey and Pothos's (2008) StimSelect software package to provide a fast, easy way for presenting AGL charts in a computerized manner and to provide commands for extracting grammatical sequences out of charts. Bollt and Jones (2000) claimed that if there is a transition matrix representing an AGL chart, then the chart's TE will be the natural logarithm of that matrix's largest non-negative eigenvalue. As detailed in Appendix B in Supplementary Material, we next wrote code extension to recover the Markov transition matrix representing the chart with the correct lift size; thus, after easily finding the eigenvalue we calculated the *TE* value. Appendix C in Supplementary Material presents an illustration of calculating TE for Reber's (1967) grammatical chart, according to Bollt and Jones's (2000) matrix-lift-action method using Bailey and Pothos's (2008) StimSelect software and our code extension.

### Data analysis

To examine the hypothesis that learners' accuracy of test performance in each experiment would be associated with the complexity level of that study's AGL chart, we first calculated the TE for each of the 10 grammar charts and then we arranged all the publications into Table 1 by grouping them according to chart. To compare learners' performance to each chart's *TE* values, we performed two Pearson correlations. One correlation was at the chart level: For each of the 10 AGL charts, we computed the correlation between TE and the mean accuracy level obtained by participants for all experiments based on that particular chart. The other correlation was at the study level, using accuracy data from the separate studies. In addition, we conducted a meta regression analysis of effect sizes, to examine whether sample size and percentage of success would predict the chart's complexity level and the participants' mean performance accuracy. In this regression, the weight of each study equaled the inverse of the variance (i.e., high variance received low weight, and vice versa). Finally, to examine age effects, we conducted analysis of variance (ANOVA) with age as an independent variable and then conducted the same analysis with TE entered as a covariate to determine whether age effects would be evident beyond the effects of grammatical complexity.

## Results

The findings of the present meta-analysis demonstrate, for the first time, that the complexity effect remains across the different settings and conditions in which the categorization task takes place. The descriptive statistics in Table 1 reveal that the *TE* value has a short range from 0.56 (low complexity) to 0.916 (high complexity), with learners' performance accuracy ranging from 47 to 75%. Yet, there is a highly significant negative Pearson correlation between the *TE* values and performance on AGL tasks. Specific examples of experiments' outcomes seem to suggest that the complexity of the AGL system as measured by TE may be related to participants' performance on the categorization task (into grammatical vs. ungrammatical categories) at the testing phase, indicating that learners may have been affected by the grammar system's difficulty. For example, in the experiments described in Witt and Vinter (2011a) and Newell and Bright (2003), the *TE* values were very high (0.916 for Grammar J and 0.856 for Grammar I, respectively, as seen on Table 1), indicating exceedingly complex grammars, and participants' task performance was only at or slightly above chance level (50 and 53.1% accuracy, respectively). In contrast, in studies with low *TE* values, the high percentage of participants' correct responses may be explained by the grammar's simplicity. To illustrate, the TEs of the grammar systems were only 0.56 in Scott and Dienes (2008; Grammar A) and 0.578 in Domangue et al. (2004; Grammar B), and participants in these studies achieved impressively high percentages of accurate responses: 69 and 70%, respectively.

In addition, highly significant negative Pearson correlations emerged between *TE* values and performance on AGL tasks, both at the chart level using the averaged accuracies for all experiments using a particular chart (*r* = −0.40, *N* = 56, *p* = 0.002), and at the study level, using accuracy data from the separate studies (*r* = −0.85, *N* = 10, *p* = 0.002). However, the outcomes of the meta regression analysis yielded a much lower, albeit significant, correlation between the grammatical complexity level and participants' mean performance accuracy in past studies: The Pearson correlation for accuracy vs. TE on corrected data was *R* = −0.316, *N* = 56, *p* = 0.013. In addition, the meta regression performed for performance accuracy included two independent variables: *TE* values, and number of stimuli (the amount of test items or strings). The general model was significant, *R* = 0.327, *N* = 56, *p* = 0.0001, and a significant negative effect of TE emerged on accuracy, *p* = 0.0001, whereas no significant effect emerged for number of stimuli on accuracy, *p* > 0.05.

With regard to the issue of age, we first grouped the surveyed studies according to child vs. adult participants in the different experiments, and we examined the *TE* values and performance levels (Table 2). Descriptive findings for children may add data about this pattern of relations between task performance and grammar complexity. To illustrate, Witt and Vinter (2011a,b) found that 5- to 7-year-olds were not successful AGL learners inasmuch as their performance rates were at guessing level (50% and less); however, these children were using the chart with the highest complexity that we measured (Grammar J: *TE* = 0.916). Conversely, Reber (1967) reported a higher percentage of correct responses in children (65.2%) when using one of the least complex charts that we measured (Grammar C: *TE* = 0.602). These discrepancies indicate that part of the reason why children score lower than adults on AGL tasks may be linked to the complexity level of the tested AGL system. We next conducted an ANOVA for performance accuracy, with age (children vs. adults) as the independent variable, and a significant age effect emerged, *F*_(1, 36)_ = 4.01, *p* = 0.05. However, when the grammatical chart's *TE* value study was entered as a covariate in an ANCOVA, the age effect was no longer significant, *F*_(1, 35)_ = 2.5, *p* = 0.12, indicating that the complexity of the AGL charts used in experimental studies might be a confounding variable with participants' age. Thus, although it was reasonable to assume that children's lower accuracy in AGL tasks compared to adults should be attributed to their younger age, the ANCOVA suggests that charts' complexity is a better predictor of accuracy than age.

**Table 2:** **Studies' distribution by participants' age**.

	**Publication year**	**AGL graph**	**Population mean age**	***TE***	**Accuracy (%)**
Scott and Dienes	2008	Reber1969	22	0.56	69.00
Kirkhart	2001	Reber1969	Students	0.56	61.40
Domangue et al.	2004	Mathews et al., 1989	Students	0.578	70.00
Reber	1967	Reber1967	Students	0.602	73.50
Reber	1967	Reber1967	Children	0.602	65.20
Zizak and Reber	2004	Reber1967	Students	0.602	62.00
Rosas et al.	2010	Reber1967	7.31	0.602	47.00
Zizak and Reber	2004	Reber1967	Students	0.602	62.00
Zizak and Reber	2004	Reber1967	Students	0.602	58.00
Zizak and Reber	2004	Reber1967	Students	0.602	59.00
Servan-Schreiber and Anderson	1990	Reber1967	Students	0.602	68.90
Poznanski and Tzelgov	2010	Reber1967	Students	0.602	67.50
Conway and Christiansen	2006	Reber1967	Students	0.602	62.00
Skosnik et al.	2002	Skosnik2002	Students	0.603	65.10
Skosnik et al.	2002	Skosnik2002	Students	0.603	57.40
Peigneux et al.	1999	Meulemans1997	63	0.686	56.13
Danion et al.	2001	Meulemans1997	33.7	0.686	74.80
Conway and Christiansen	2006	Conway2006	Students	0.716	66.00
Conway and Christiansen	2006	Conway2006	Students	0.716	58.00
Knowlton and Squire	1996	Konwlton1996	63.8	0.74	63.50
Pavlidou et al.	2009	Konwlton1996	6.48	0.74	59.37
Chang and Knowlton	2004	Konwlton1996	Students	0.74	62.50
Pavlidou et al.	2010	Konwlton1996	9.3	0.74	55.00
Chang and Knowlton	2004	Konwlton1996	Students	0.74	64.70
Pavlidou and Williams	2010	Konwlton1996	Children up to 12	0.74	60.00
Don et al.	2003	Konwlton1996	23	0.74	66.00
Pothos and Kirk	2004	Konwlton1996	Students	0.74	49.00
Pothos et al.	2006	Konwlton1996	Students	0.74	67.20
Pothos et al.	2006	Konwlton1996	Students	0.74	64.80
Horan et al.	2008	Konwlton1996	18–25	0.74	58.00
Pothos and Bailey	2000	Konwlton1996	Students	0.74	59.00
Pothos et al.	2006	Reber1978	Students	0.761	55.40
Pothos et al.	2006	Reber1978	Students	0.761	53.30
Pothos	2005	Reber1978	Students	0.761	61.87
Rüsseler et al.	2006	Reber1978	32.75	0.761	64.10
Perruchet and Pacteau	1990	Reber1978	Students	0.761	63.30
Reber and Perruchet	2003	Reber1978	Students	0.761	55.60
Reber and Perruchet	2003	Reber1978	Students	0.761	56.20
Reber and Perruchet	2003	Reber1978	Students	0.761	55.30
Smith et al.	2001	Reber1978	68.36	0.761	57.90
de Vries et al.	2009	Reber1978	22.6	0.761	66.40
Jamieson and Mewhort	2010	Reber1978	Students	0.761	60.00
Jamieson and Mewhort	2010	Reber1978	Students	0.761	63.00
Knowlton and Squire	1994	Brooks1991	62.4	0.856	62.50
Gebauer and Mackintosh	2007	Brooks1991	11–32	0.856	66.42
Knowlton and Squire	1994	Brooks1991	64.9	0.856	60.90
Meulemans and Van der Linden	1997	Brooks1991	Students	0.856	55.60
Meulemans and Van der Linden	1997	Brooks1991	Students	0.856	54.10
Higham	1997b	Brooks1991	Students	0.856	58.00
Tunney	2005	Brooks1991	19.42	0.856	58.00
Newell and Bright	2003	Brooks1991	Students	0.856	51.30
Higham	1997a	Brooks1991	Students	0.856	64.00
Higham	1997a	Brooks1991	Students	0.856	50.00
Higham	1997a	Brooks1991	Students	0.856	52.00
Witt and Vinter	2011a	Witt2011	5–7	0.916	50.00
Witt and Vinter	2011b	Witt2011	5–7	0.916	50.00

## Discussion

The present study has enhanced the usefulness and practicality of TE, the AGL complexity measure introduced by Bollt and Jones (2000), by automating the process of obtaining *TE* values from memoryless charts. This new matrix-lift-action method enables uniform comparisons among different experimental research publications that previously utilized varying charts for AGL testing. By reviewing previously published AGL experiments, this study redefines the significance of TE as a measure of grammar complexity and provides researchers with an efficient means for calculating the complexity of a given AGL system. Furthermore, the current meta-analysis documenting diverse charts' range of *TE* values pinpoints complexity level as an important measurement to be taken into account by future researchers when designing and selecting their experimental AGL stimuli.

The current findings validate and extend the existing literature investigating learners' performance on tasks of varying complexity. Evidence is available demonstrating that higher *TE* values coincide with poorer ability to categorize test items as grammatical or ungrammatical, and vice versa (e.g., Van den Bos and Poletiek, 2008); however, such research tested the same group of participants on AGL systems of different levels of difficulty. The current study adopted a broader approach by systematically surveying the literature to locate previous AGL experiments that were carried out under different conditions, and by using meta-regression analysis to establish that the TE measure significantly correlates with performance across AGL tasks. Future researchers use the matrix-lift-action method to compare implicit vs. explicit learning conditions as well as different complexity levels in studies with children. Other complexity measures (such as the redundancy method, TRS, and the bigram/ trigram method) which refer to the other components of the AGL task might also play a role. It is recommended that future studies examine the correlation of these metrics with respect to the performance data, comparing TE with them.

## Concluding remarks

Complexity has been previously shown to affect performance on artificial grammar learning (AGL) tasks. Yet, past AGL studies did not employ consistent measures to examine the comprehensive effect of grammar complexity on task performance. In the present study we computerized Bollt and Jones's (2000) technique of calculating topological entropy (TE), a quantitative measure of AGL charts' complexity, with the aim of examining associations between grammar systems' TE and learners' AGL task performance. The results of the meta-regression analysis indicate that the complexity effect transcended the different settings and conditions in which the categorization task was performed. The results reinforced the significance of utilizing this new automated tool to uniformly measure grammar systems' complexity when experimenting with and evaluating the findings of AGL studies.

### Conflict of interest statement

The authors declare that the research was conducted in the absence of any commercial or financial relationships that could be construed as a potential conflict of interest.

## References

[B1] Andrade J. Baddeley A. (2011). The contribution of phonological short-term memory to artificial grammar learning. Q. J. Exp. Psychol. 64, 960–974 10.1080/17470218.2010.533440 21287426

[B2] Aslin R. N. Newport E. L. (2008). What statistical learning can and can't tell us about language acquisition, in Infant Pathways to Language: Methods, Models, and Research Directions, eds Colombo J. McCardle P. Freund L. (Mahwah, NJ: Erlbaum), 15–29

[B3] Bailey T. M. Pothos E. M. (2008). AGL StimSelect: Software for automated selection of stimuli for artificial grammar learning. Behav. Res. Methods 40, 164–176 10.3758/BRM.40.1.164 18411539

[B4] Bollt E. M. Jones M. A. (2000). The complexity of artificial grammars. Nonlin. Dyn. Psychol. Life Sci. 4, 153–168 10.1023/A:1009524428448

[B5] Brooks L. R. Vokey J. R. (1991). Abstract analogies and abstracted grammars: Comments on Reber (1989). J. Exp. Psychol. 120, 316–323 10.1037/0096-3445.120.3.316

[B6] Chang G. Y. Knowlton B. J. (2004). Visual feature learning in artificial grammar classification. J. Exp. Psychol. 30, 714–722 10.1037/0278-7393.30.3.714 15099138

[B7] Cleeremans A. Destrebecqz A. Boyer M. (1998). Implicit learning: news from the front. Trends Cogn. Sci. 2, 406–416 10.1016/S1364-6613(98)01232-7 21227256

[B8] Conway C. M. Christiansen M. H. (2006). Statistical learning within and between modalities pitting abstract against stimulus-specific representations. Psychol. Sci. 17, 905–912 10.1111/j.1467-9280.2006.01801.x 17100792

[B9] Danion J. M. Meulemans T. Kauffmann-Muller F. Vermaat H. (2001). Intact implicit learning in schizophrenia. Am. J. Psychiatry 158, 944–948 10.1176/appi.ajp.158.6.944 11384904

[B10] de Vries M. H. Barth A. C. R. Maiworm S. Knecht S. Zwitserlood P. Flöel A. (2009). Electrical stimulation of Broca's area enhances implicit learning of an artificial grammar. J. Cogn. Neurosci. 22, 2427–2436 10.1162/jocn.2009.21385 19925194

[B11] de Vries M. H. Ulte C. Zwitserlood P. Szymanski B. Knecht S. (2010). Increasing dopamine levels in the brain improves feedback-based procedural learning in healthy participants: an artificial-grammar-learning experiment. Neuropsychologia 48, 3193–3197 10.1016/j.neuropsychologia.2010.06.024 20600185

[B12] Dienes Z. Broadbent D. Berry D. (1991). Implicit and explicit knowledge bases in artificial grammar learning. J. Exp. Psychol. 17, 875–887 10.1037/0278-7393.17.5.875 1834769

[B13] Domangue T. J. Mathews R. C. Sun R. Roussel L. G. Guidry C. E. (2004). Effects of model-based and memory-based processing on speed and accuracy of grammar string generation. J. Exp. Psychol. 30, 1002–1011 10.1037/0278-7393.30.5.1002 15355132

[B14] Don A. J. Schellenberg E. G. Reber A. S. DiGirolamo M. K. Wang P. P. (2003). Implicit learning in children and adults with Williams Syndrome. Dev. Neuropsychol. 23, 201–225 10.1080/87565641.2003.9651892 12730025

[B15] Dulany D. E. Carlson R. A. Dewey G. I. (1984). A case of syntactical learning and judgment: how conscious and how abstract? J. Exp. Psychol. 113, 541–555 10.1037/0096-3445.113.4.541

[B16] Endress A. D. Bonatti L. L. (2007). Rapid learning of syllable classes from a perceptually continuous speech stream. Cognition 105, 247–299 10.1016/j.cognition.2006.09.010 17083927

[B17] Evans J. L. Saffran J. R. Robe-Torres K. (2009). Statistical learning in children with specific language impairment. J. Speech Lang. Hear. Res. 52, 321–335 10.1044/1092-4388(2009/07-0189) 19339700 PMC3864761

[B18] Gebauer G. F. Mackintosh N. J. (2007). Psychometric intelligence dissociates implicit and explicit learning. J. Exp. Psychol. 33, 34–54 10.1037/0278-7393.33.1.34 17201553

[B19] Higham P. A. (1997a). Chunks are not enough: the insufficiency of feature frequency-based explanations of artificial grammar learning. Can. J. Exp. Psychol. 51, 126–137 10.1037/1196-1961.51.2.126

[B20] Higham P. A. (1997b). Dissociations of grammaticality and specific similarity effects in artificial grammar learning. J. Exp. Psychol. 23, 1029–1045 10.1037/0278-7393.23.4.1029 9231440

[B21] Horan W. P. Green F. M. Knowlton B. J. Wynn J. K. Mintz J. Nuechterlein K. H. (2008). Impaired implicit learning in Schizophrenia. Neuropsychology 22, 606–617 10.1037/a0012602 18763880 PMC2548320

[B22] Jamieson R. K. Mewhort D. J. K. (2005). The influence of grammatical, local, and organizational redundancy on implicit learning: an analysis using information theory. J. Exp. Psychol. 31, 9–23 10.1037/0278-7393.31.1.9 15641901

[B23] Jamieson R. K. Mewhort D. J. K. (2010). Applying an exemplar model to the artificial-grammar task: string completion and performance on individual items. Q. J. Exp. Psychol. 63, 1014–1039 10.1080/17470210903267417 19851941

[B24] Johnstone T. Shanks D. R. (1999). Two mechanisms in implicit artificial grammar learning? Comment on Meulemans and Van der Linden (1997). J. Exp. Psychol. 25, 524–531 10.1037/0278-7393.25.2.524

[B25] Johnstone T. Shanks D. R. (2001). Abstractionist and processing accounts of implicit learning. Cogn. Psychol. 42, 61–112 10.1006/cogp.2000.0743 11161417

[B26] Kirkhart M. W. (2001). The nature of declarative and nondeclarative knowledge for implicit and explicit learning. J. Gen. Psychol. 128, 447–461 10.1080/00221300109598921 11892891

[B27] Knowlton B. J. Squire L. R. (1994). The information acquired during artificial grammar learning. J. Exp. Psychol. 20, 79–91 10.1037/0278-7393.20.1.79 8138790

[B28] Knowlton B. J. Squire L. R. (1996). Artificial grammar learning depends on implicit acquisition of both abstract and exemplar-specific information. J. Exp. Psychol. Learn. Mem. Cogn. 22, 169–181 10.1037/0278-7393.22.1.169 8648284

[B29] Lorsbach T. Worman L. J. (1989). The development of explicit and implicit forms of memory in learning disabled children. Contemp. Educ. Psychol. 14, 67–76 10.1016/0361-476X(89)90006-4

[B30] Marcus G. F. Brinkmann U. Clahsen H. Wiese R. Pinker S. (1995). German inflection: the exception that proves the rule. Cogn. Psychol. 29, 189–256 10.1006/cogp.1995.1015 8556846

[B31] Mathews R. C. Buss R. R. Stanley W. B. Blanchard-Fields F. Cho J. R. Druhan B. (1989). Role of implicit and explicit processes in learning from examples: a synergistic effect. J. Exp. Psychol. 15, 1083–1110 10.1037/0278-7393.15.6.1083

[B32] Meulemans T. Van der Linden M. (1997). Associative chunk strength in artificial grammar learning. J. Exp. Psychol. 23, 1007–1028 10.1037/0278-7393.23.4.1007

[B33] Newell B. R. Bright J. E. H. (2003). The subliminal mere exposure effect does not generalize to structurally related stimuli. Can. J. Exp. Psychol. 57, 61–68 10.1037/h0087413 12674370

[B34] Pavlidou E. V. Kelly L. M. Williams J. M. (2010). Do children with developmental dyslexia have impairments in implicit learning? Dyslexia 16, 143–161 10.1002/dys.400 20440744

[B35] Pavlidou E. V. Williams J. M. (2010). Developmental dyslexia and implicit learning: evidence from an AGL transfer study. Procedia Soc. Behav. Sci. 2, 3289–3296 10.1016/j.sbspro.2010.03.503

[B36] Pavlidou E. V. Williams J. M. Kelly L. M. (2009). Artificial grammar learning in primary school children with and without developmental dyslexia. Ann. Dyslexia 59, 55–77 10.1007/s11881-009-0023-z 19326218

[B37] Peigneux P. Meulemans T. Ven der Linden M. Salmon E. Petit H. (1999). Exploration of implicit artificial grammar learning in Parkinson's disease. Acta Neurol. Belg. 99, 107–117 10427353

[B38] Pena M. Bonatti L. L. Nespor M. Mehler J. (2002). Signal-driven computations in speech processing. Science 298, 604–607 10.1126/science.1072901 12202684

[B39] Perruchet P. Pacteau C. (1990). Synthetic grammar learning: Implicit rule abstraction or explicit fragmentary knowledge? J. Exp. Psychol. 119, 264–275 10.1037/0096-3445.119.3.264

[B40] Perruchet P. Vinter A. S. Michael A. (1998). Learning and development: the implicit knowledge assumption reconsidered, in Handbook of implicit learning, ed Frensch P. A. (Thousand Oaks, CA: Sage), 495–531

[B41] Pothos E. M. (2005). Expectations about stimulus structure in implicit learning. Mem. Cogn. 33, 171–181 10.3758/BF03195306 15915802

[B42] Pothos E. M. (2010). An entropy model for artificial grammar learning. Front. Psychol. 1:16 10.3389/fpsyg.2010.00016 21607072 PMC3095384

[B43] Pothos E. M. Bailey T. M. (2000). The importance of similarity in artificial grammar learning. J. Exp. Psychol. Learn. Mem. Cogn. 26, 847–862 10.1037/0278-7393.26.4.847 10946367

[B44] Pothos E. M. Chater N. Ziori E. (2006). Does stimulus appearance affect learning? Am. J. Psychol. 119, 277–301 10.2307/20445339 16841782

[B45] Pothos E. M. Kirk J. (2004). Investigating learning deficits associated with dyslexia. Dyslexia 10, 61–76 10.1002/dys.266 14998143

[B46] Poznanski Y. Tzelgov J. (2010). Modes of knowledge acquisition and retrieval in artificial grammar learning. Q. J. Exp. Psychol. 63, 1495–1515 10.1080/17470210903398121 20063258

[B47] Reber A. S. (1967). Implicit learning of artificial grammars. J. Verbal Learn. Verbal Behav. 6, 855–863 10.1016/S0022-5371(67)80149-X

[B48] Reber A. S. (1969). Transfer of syntactic structure in synthetic languages. J. Exp. Psychol. 81, 115–119 10.1037/h0027454

[B49] Reber A. S. (1989). Implicit learning and tacit knowledge. J. Exp. Psychol. 118, 219–235 10.1037/0096-3445.118.3.219

[B51] Reber P. J. Squire L. R. (1999). Intact learning of artificial grammars and intact category learning by patients with Parkinson's disease. Behav. Neurosci. 113, 235–242 10.1037/0735-7044.113.2.235 10357448

[B52] Reber R. Perruchet P. (2003). The use of control groups in artificial grammar learning. Q. J. Exp. Psychol. 56A, 97–115 10.1080/02724980244000297 12587897

[B53] Redington M. Chater N. (1996). Transfer in artificial grammar learning: a reevaluation. J. Exp. Psychol. 125, 123–138 10.1037/0096-3445.125.2.123

[B54] Rohrmeier M. Rebuschat P. Cross I. (2011). Incidental and online learning of melodic structure. Conscious. Cogn. 20, 214–222 10.1016/j.concog.2010.07.004 20832338

[B55] Rosas R. Ceric F. Tenorio M. Mourgues C. Thibaut C. Hurtado E. (2010). ADHD children outperform normal children in an artificial grammar implicit learning task: ERP and RT evidence. Conscious. Cogn. 19, 341–351 10.1016/j.concog.2009.09.006 20116292

[B56] Rüsseler J. Gerth I. Münte M. F. (2006). Implicit learning is intact in adult developmental dyslexic readers: evidence from serial reaction time task and artificial grammar learning. J. Clin. Exp. Neuropsychol. 28, 808–827 10.1080/13803390591001007 16723326

[B57] Saffran J. R. Aslin R. N. Newport E. L. (1996). Statistical learning by 8-month-old infants. Science 274, 1926–1928 10.1126/science.274.5294.1926 8943209

[B58] Saffran J. R. Newport E. L. Aslin R. N. Tunick R. A. Barrueco S. (1997). Incidental language learning: listening (and learning) out of the corner of your ear. Psychol. Sci. 8, 101–105 10.1111/j.1467-9280.1997.tb00690.x10193055

[B59] Scott R. B. Dienes Z. (2008). The conscious, the unconscious, and familiarity. J. Exp. Psychol. 34, 1264–1288 10.1037/a0012943 18763904

[B60] Servan-Schreiber E. Anderson J. R. (1990). Learning artificial grammars with competitive chunking. J. Exp. Psychol. 16, 592–608 10.1037/0278-7393.16.4.59224905545

[B61] Skosnik P. D. Mirza F. Gitelman D. R. Parrish T. B. Mesulam M.-M. Reber P. J. (2002). Neural correlates of artificial grammar learning. Neuroimage 17, 1306–1314 10.1006/nimg.2002.1291 12414270

[B62] Smith J. G. Siegert R. J. McDowall J. Abernethy D. (2001). Preserved implicit learning on both the serial reaction time task and artificial grammar in patients with Parkinson's disease. Brain Cogn. 45, 378–391 10.1006/brcg.2001.1286 11305880

[B63] Tunney R. J. (2005). Sources of confidence judgments in implicit cognition. Psychon. Bull. Rev. 12, 367–373 10.3758/BF03196386 16082820

[B64] Van den Bos E. Poletiek F. H. (2008). Effects of grammar complexity on artificial grammar learning. Mem. Cogn. 36, 1122–1131 10.3758/MC.36.6.1122 18927030

[B65] Witt A. Vinter A. (2011a). Artificial grammar learning in children: abstraction of rules or sensitivity to perceptual features? Psychol. Res. 76, 97–110 10.1007/s00426-011-0328-5 21437612

[B66] Witt A. Vinter A. (2011b). Learning implicitly to produce avoided behaviors. Q. J. Exp. Psychol. 1–14 10.1080/17470218.2010.543283 21391157

[B67] Ziegler J. C. Goswami U. (2005). Reading acquisition, developmental dyslexia, and skilled reading across languages: a psycholinguistic grain size theory. Psychol. Bull. 131, 3–29 10.1037/0033-2909.131.1.3 15631549

[B68] Zizak D. M. Reber A. S. (2004). Implicit preferences: the role(s) of familiarity in the structural mere exposure effect. Conscious. Cogn. 13, 336–362 10.1016/j.concog.2003.12.003 15134764

