# Supplementary material for: Does complexity matter? Meta-analysis of learner performance in artificial grammar tasks
Source: Front Psychol. 2014 Sep 25;5:1084. doi: 10.3389/fpsyg.2014.01084 (PMC4174743; doi:10.3389/fpsyg.2014.01084)
Supplement: Supplementary file 1 [file DataSheet1.PDF]

Appendix A

Comparing Direct Formula Versus Bollt & Jones' (2000) Method of Calculating Topographical Entropy (TE)

We use the following example of a simple chart to emphasize the difficulty in calculating TE using the direct formula as compared to Bollt and Jones's (2000) method:

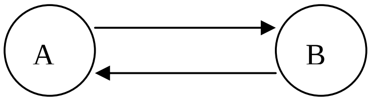

Direct Formula

The direct formula for calculating the TE is: $h(\Sigma_M) = \lim_{n\to\infty}\left[\frac{\ln(w_n(\Sigma_M))}{n}\right]$

We will now find the values in the formula for the simple chart above. For a 1-letter word (*n* = 1), Σ_M is a closed group A and B, so its size is $w_1(\Sigma_M)$=2. For *n* = 2, Σ_M is a closed group AB and BA, so its size is $w_2(\Sigma_M)$=2. The table below demonstrates how for different word lengths (*n*), Σ_M remains a closed group containing only two possible words:

n	$\sum_M$	$W_n(\sum_M)$
1	A,B	2
2	AB, BA	2
3	ABA, BAB	2
$n \to \infty$	ABAB, BABA,...	2

We will then list all the components in the formula:

$$TE = h(\Sigma_M) = \lim_{n\to\infty}\left[\frac{\ln(w_n(\Sigma_M))}{n}\right] = \lim_{n\to\infty}\left[\frac{\ln(2)}{n}\right] = 0$$

Bollt & Jones's (2000) Method

We now calculate TE for the same simple chart using Bollt and Jones's method. First we build a matrix:

	**A**	**B**
**A**	0	1
**B**	1	0

Using the computer, we can now calculate the maximum eigenvalue: Max(Eig[Matrix]) = 1

We next calculate TE, which is ln(1) = 0. We can see that in both instances we obtain the same results.

Applying Both Calculation Methods to a Complex Chart

Now we will apply both calculation methods to a previously used AGL chart, that of Reber from 1967 (shown also in Grammar C on Figure 1):

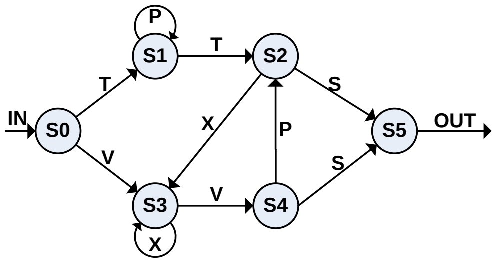

First, we apply the direct formula, as seen in the next table. Note that calculating $n \to \infty$ is very complicated and may be impossible:

n	$\sum_M$	$W_n\left(\sum_M\right)$
2	TP, TT, VX, VV, PP, PT, XX, XV…	17
3	TPP, TPT, VXX, VXV, PPP, PPT, PTS, PTX…	31
4	TPPP, TPPT, TPTS, TPTX, TTXX…	57
5	TPPPP, TPPPT, VXXXX, VXXXV…	104
6	TPPPPP, TPPPPT, VXXXXX, VXXXXV…	190
$n \to \infty$	Infinite group of symbols	∞

Next, we apply Bollt and Jones's (2000) method. We start by building a matrix for Reber's complex chart. This matrix must be derived from a memoryless system, and because all of this chart's symbols (letters) appear more than once, we need to use pairs of letters. This process, defined above as "lift action," may be needed in more complex charts where more than 2 letters may be required to define a unique state in the chart (which can be done manually or by a computer). We place all possible pairs in the matrix as shown below, and then we map all of the chart's transitions by placing 1 in the matrix for every grammatical transition (that follows the chart's rules) and 0 for every ungrammatical transition. Note that in this method we need to consider the chart to be self-linked (output goes to input) because we need to be able to create endless words. We represent the transitions between output and input with the symbol "_."

	PP	PS	PT	PX	S _	TP	TS	TT	TX	VP	VS	VV	VX	XV	XX	_T	_V
PP	1	0	1	0	0	0	0	0	0	0	0	0	0	0	0	0	0
PS	0	0	0	0	1	0	0	0	0	0	0	0	0	0	0	0	0
PT	0	0	0	0	0	0	1	0	1	0	0	0	0	0	0	0	0
PX	0	0	0	0	0	0	0	0	0	0	0	0	0	1	1	0	0
S_	0	0	0	0	0	0	0	0	0	0	0	0	0	0	0	1	1
TP	1	0	1	0	0	0	0	0	0	0	0	0	0	0	0	0	0
TS	0	0	0	0	1	0	0	0	0	0	0	0	0	0	0	0	0
TT	0	0	0	0	0	0	1	0	1	0	0	0	0	0	0	0	0
TX	0	0	0	0	0	0	0	0	0	0	0	0	0	1	1	0	0
VP	0	1	0	1	0	0	0	0	0	0	0	0	0	0	0	0	0
VS	0	0	0	0	1	0	0	0	0	0	0	0	0	0	0	0	0
VV	0	0	0	0	0	0	0	0	0	1	1	0	0	0	0	0	0
VX	0	0	0	0	0	0	0	0	0	0	0	0	0	1	1	0	0
XV	0	0	0	0	0	0	0	0	0	1	1	0	0	0	0	0	0
XX	0	0	0	0	0	0	0	0	0	0	0	0	0	1	1	0	0
_T	0	0	0	0	0	1	0	1	0	0	0	0	0	0	0	0	0
_V	0	0	0	0	0	0	0	0	0	0	0	1	1	0	0	0	0

Using the computer, we can now calculate the maximum eigenvalue and the TE: Max(Eig[Matrix]) = 1.826; TE = ln(1.826) = 0.602. As seen, Bollt and Jones's method for calculating TE for a chart is much more practical than using a direct calculation from the formula.

Appendix B

Implementing Bollt and Jones' (2000) theory with Matlab

As seen in Appendix A, Bollt and Jones (2000) set up a practical method for TE calculation using a Markov matrix that represents the AGL chart. The key is to build the right matrix and to ensure that it represents a memoryless system. Inasmuch as the matrix may contain dozens of lines, calculating TE manually may be inordinately time-consuming and may lead to inaccuracies. To efficiently and accurately build the matrix, we needed to create a compact software program with two main functions: generating all of a grammar chart's strings and building a transition matrix from those strings. To generate the strings, we used Bailey and Pothos's (2008) StimSelect software, which they created to automatically generate training and testing strings. The code for the software appears on their site and is explained in their article.

Unfortunately, StimSelect does not offer any way to extract a Markov transition matrix that represents the chart; therefore, to build a transition matrix from the grammatical strings, we wrote a software code extension to StimSelect as follows. Knowing that the matrix is the key to the connections between all strings of size *n* (the minimum size of a word, equaling 1), we found all strings of size *n* that were grammatical and placed them on the matrix. Next, to identify which strings were connected, we found all grammatical strings of size M = *n*+1. Knowing that every grammatical string of size *n*+1 consists of two grammatical sub-strings of size *n*, we found the connections between all strings of size *n*. For example, if the sequence ABCD is grammatical, there must be a connection between the two sub-strings (trigrams) ABC and BCD, and in the matrix we can insert "1" in the cell marking their intersection. We established the following **algorithm** in order to build the matrix for string size *n*:

1. Find all grammatical sequences of size *n* and place them in an array, using StimSelect.
2. Find all grammatical sequences of size M (defined as M=*n*+1), using StimSelect.
3. For each sequence of size M:
   a. Find the first sequence of size *n*.
   b. Find the second sequence of size *n*.
   c. Column is the position of the first sequence in the array.
   d. Row is the position of the second sequence in the array.
   e. Mark the transition between the first and second sequences by placing "1" in the correct column and row.

Commands used:

To find all strings (Str) of size M in the grammar chart (similar is for size *n*):

Str_M = grammatical_strings(grammar,[M M],1:n_states)

To obtain first sequence of size *n* from Str_M:

sub_1 = Str_M(1:M-1)

To obtain second sequence of size *n* from Str_M:

sub_2 = Str_M(2:M)

To mark a transition in matrix M:

M(column, row) = 1

Once we built the transition matrix correctly, we needed to find a sufficient lift for a memoryless representation of the chart. Embracing Theorem 6 in Bollt and Jones (2000), “If M and N are transition matrices of Markov representations of the same artificial grammar, then their topological entropies are equal,” this means that if M and *n* are generated by the same chart and their TEs are equal, then this is the chart's TE. Thus, we generated a matrix from the same artificial grammar with growing lift order (larger string size) until we found two matrices with same largest nonnegative eigenvalue, using the eig(M) command.

Appendix C

Sample Application of TE Calculation Using StimSelect

To illustrate TE calculation according to Bollt and Jones's (2000) matrix-lift-action method by means of Bailey and Pothos's (2008) StimSelect software, we return to Reber's (1967) chart:

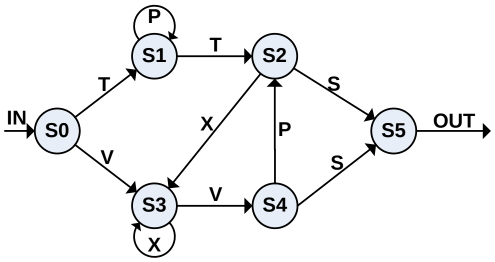

1. We define this chart in StimSelect with two basic assumptions:
   a. Stimuli can end at any state (defined by StimSelect software as symbol '.' goes to State -1)
   b. Transition from S6 to S1 generates the symbol '_'

```
function [g n_states] = Reber1967( )
clear
'Reber1967'
syms = 'TVPXS_';% Symbols accepted by this grammar
n_states = 6; % Number of states in the grammar
g = fsg([], syms, n_states);% Create an FSG (finite state grammar) object for these symbols
g = link(g, 1, 'TV.', [2 3 -1]);
g = link(g, 2, 'PT.', [2 4 -1]);
g = link(g, 3, 'XV.', [3 5 -1]);
g = link(g, 4, 'XS.', [3 6 -1]);
g = link(g, 5, 'PS.', [4 6 -1]);
g = link(g, 6, '_.', [1 -1]);
return
```

2. Building transition matrix without lift (n=1)
   a. Find all strings in size 1 and all strings in size 2

```
str_1 = grammatical_strings(grammar, [1 1], 1:n_states);
str_2 = grammatical_strings(grammar, [2 2], 1:n_states);
```

str_1 = P S T V X _

str_2 = PP PS PT PX S_ TP TS TT TX VP VS VV VX XV XX _T _V

b. Find all transitions in str_2 and put them in matrix_1

```
for index = 1:len_2
 sub_1 = str_2(index,1);
 sub_2 = str_2(index,2);
 posX= strcmp(cell_1,sub_1);
 posY = strcmp(cell_1,sub_2);
 Matrix_1(posX,posY) = 1;
end
```

PP: P -> P

PS: P -> S

PT: P -> T

PX: P -> X

S_: S -> _

TP: T -> P

TS: T -> S

TT: T -> T

TX: T -> X

VP: V -> P

VS: V -> S

VV: V -> V

VX: V -> X

XV: X -> V

XX: X -> X

_T: _ -> T

_V: _ -> V

	P	S	T	V	X	_
P	1	1	1	0	1	0
S	0	0	0	0	0	1
T	1	1	1	0	1	0
V	1	1	0	1	1	0
X	0	0	0	1	1	0
_	0	0	1	1	0	0

3. Calculate TE candidate for the matrix (We see this TE as a candidate until we find TE from a higher lift that matches this TE).

```
Eigen=eig(Matrix_1);
maxEig = max(Eigen);
TE(1) = log(maxEig);
```

maxEig = 2.8072

TE(1) = 1.0322

4. Building transition matrix with minimum lift (*n*=2)
    a. Find all strings in size 2 and all strings in size 3

    str_2 = grammatical_strings(grammar, [2 2], 1:n_states);

    str_3 = grammatical_strings(grammar, [3 3], 1:n_states);

    str_2 = PP PS PT PX S_ TP TS TT TX VP VS VV VX XV XX _T _V

    str_3 = PPP PPT PS_ PTS PTX PXV PXX S_T S_V TPP TPT TS_ TTS TTX TXV TXX VPS VPX VS_ VVP VVS VXV VXX XVP XVS XXV XXX _TP _TT _VV _VX

    b. Find all transitions in str_3 and put them in matrix_2

    for index = 1:len_3

    sub_1 = str_3(index,1:2);%first bigram in this trigram

    sub_2 = str_3(index,2:3);%second bigram in this trigram

    posX= strcmp(cell_2,sub_1);%find the position in matrix of the first bigram

    posY = strcmp(cell_2,sub_2);%find the position in matrix of the second bigram

    Matrix_2(posX,posY) = 1;

    end

PPP: PP -> PP

PPT: PP -> PT

PS_: PS -> S_

PTS: PT -> TS

PTX: PT -> TX

PXV: PX -> XV

PXX: PX -> XX

S_T: S_ -> _T

S_V: S_ -> _V

TPP: TP -> PP

TPT: TP -> PT

TS_: TS -> S_

TTS: TT -> TS

TTX: TT -> TX

TXV: TX -> XV

TXX: TX -> XX

VPS: VP -> PS

VPX: VP -> PX

VS_: VS -> S_

VVP: VV -> VP

VVS: VV -> VS

VXV: VX -> XV

VXX: VX -> XX

XVP: XV -> VP

XVS: XV -> VS

XXV: XX -> XV

XXX: XX -> XX

_TT: _T -> TT

_VX: _V -> VX

_TP: _T -> TP

_VV: _V -> VV

	PP	PS	PT	PX	S	TP	TS	TT	TX	VP	VS	VV	VX	XV	XX	T	V
PP	1	0	1	0	0	0	0	0	0	0	0	0	0	0	0	0	0
PS	0	0	0	0	1	0	0	0	0	0	0	0	0	0	0	0	0
PT	0	0	0	0	0	0	1	0	1	0	0	0	0	0	0	0	0
PX	0	0	0	0	0	0	0	0	0	0	0	0	0	1	1	0	0
S	0	0	0	0	0	0	0	0	0	0	0	0	0	0	0	1	1
TP	1	0	1	0	0	0	0	0	0	0	0	0	0	0	0	0	0
TS	0	0	0	0	1	0	0	0	0	0	0	0	0	0	0	0	0
TT	0	0	0	0	0	0	1	0	1	0	0	0	0	0	0	0	0
TX	0	0	0	0	0	0	0	0	0	0	0	0	0	1	1	0	0
VP	0	1	0	1	0	0	0	0	0	0	0	0	0	0	0	0	0
VS	0	0	0	0	1	0	0	0	0	0	0	0	0	0	0	0	0
VV	0	0	0	0	0	0	0	0	0	1	1	0	0	0	0	0	0
VX	0	0	0	0	0	0	0	0	0	0	0	0	0	1	1	0	0
XV	0	0	0	0	0	0	0	0	0	1	1	0	0	0	0	0	0
XX	0	0	0	0	0	0	0	0	0	0	0	0	0	1	1	0	0
T	0	0	0	0	0	1	0	1	0	0	0	0	0	0	0	0	0
V	0	0	0	0	0	0	0	0	0	0	0	1	1	0	0	0	0

5. Calculate candidate TE for matrix

    Eigen=Eig(Matrix_2);

    maxEig = max(Eigen);

    TE(2) = log(maxEig);

    maxEig = 1.8265

    TE(2) = 0.6024

This TE does not match TE(1), so we will try higher lift.

6. In the same way, calculate the TE for matrix with higher lift (*n*=3)

    Eigen=Eig(Matrix_3);

```
maxEig = max(Eigen);
TE(3) = log(maxEig);
maxEig = 1.8265
TE(3) = 0.6024
```

This TE does match TE(2), so according to Bollt and Jones, because we found 2 matrixes that result in the same TE, and which were generated from the same chart, we can say that this is the chart's TE. In addition, we can also say that the minimum lift needed for this chart to be presented as a memoryless system is $n = 2$.
